# A deep intronic recurrent *CHEK2* variant c.1009-118_1009-87delinsC affects pre-mRNA splicing and contributes to hereditary breast cancer predisposition

**DOI:** 10.1016/j.breast.2024.103721

**Published:** 2024-03-25

**Authors:** Petra Zemankova, Marta Cerna, Klara Horackova, Corinna Ernst, Jana Soukupova, Marianna Borecka, Britta Blümcke, Leona Cerna, Monika Cerna, Vaclava Curtisova, Tatana Dolezalova, Petra Duskova, Lenka Dvorakova, Lenka Foretova, Ondrej Havranek, Jan Hauke, Eric Hahnen, Miloslava Hodulova, Milena Hovhannisyan, Lucie Hruskova, Marketa Janatova, Maria Janikova, Sandra Jelinkova, Pavel Just, Marcela Kosarova, Monika Koudova, Vera Krutilkova, Eva Machackova, Katerina Matejkova, Renata Michalovska, Adela Misove, Petr Nehasil, Barbora Nemcova, Jan Novotny, Ales Panczak, Pavel Pesek, Ondrej Scheinost, Drahomira Springer, Barbora Stastna, Viktor Stranecky, Ivan Subrt, Spiros Tavandzis, Eva Tureckova, Kamila Vesela, Zdenka Vlckova, Michal Vocka, Barbara Wappenschmidt, Tomas Zima, Zdenek Kleibl, Petra Kleiblova

**Affiliations:** a Institute of Medical Biochemistry and Laboratory Diagnostics, First Faculty of Medicine, Charles University and General University Hospital in Prague, Prague, Czech Republic; b Institute of Pathological Physiology, First Faculty of Medicine, Charles University, Prague, Czech Republic; c Center for Familial Breast and Ovarian Cancer, Center for Integrated Oncology (CIO), Medical Faculty, University of Cologne and University Hospital Cologne, Cologne, Germany; d GENNET, Prague, Czech Republic; e Institute of Medical Genetics, University Hospital Pilsen, Pilsen, Czech Republic; f Department of Medical Genetics, University Hospital Olomouc, Faculty of Medicine and Dentistry, Palacky University, Olomouc, Czech Republic; g Hospital Ceske Budejovice, Ceske Budejovice, Czech Republic; h Department of Pediatrics and Inherited Metabolic Disorders, First Faculty of Medicine, Charles University and General University Hospital in Prague, Prague, Czech Republic; i Department of Cancer Epidemiology and Genetics, Masaryk Memorial Cancer Institute, Brno, Czech Republic; j Institute of Biology and Medical Genetics, First Faculty of Medicine, Charles University and General University Hospital in Prague, Prague, Czech Republic; k BIOCEV, First Faculty of Medicine, Charles University, Prague, Czech Republic; l GHC Genetics, Prague, Czech Republic; m Pronatal, Prague, Czech Republic; n Department of Medical Genetics, AGEL Laboratories, AGEL Research and Training Institute, Novy Jicin, Czech Republic; o Department of Genetics and Microbiology, Faculty of Science, Charles University, Prague, Czech Republic; p Department of Biochemistry, Faculty of Science, Charles University, Prague, Czech Republic; q Department of Oncology, First Faculty of Medicine, Charles University and General University Hospital in Prague, Prague, Czech Republic

**Keywords:** Deep intronic *CHEK2* variant, Breast cancer, NGS, RNA analysis, Genetic testing

## Abstract

Germline *CHEK2* pathogenic variants confer an increased risk of female breast cancer (FBC). Here we describe a recurrent germline intronic variant c.1009-118_1009-87delinsC, which showed a splice acceptor shift in RNA analysis, introducing a premature stop codon (p.Tyr337PhefsTer37).

The variant was found in 21/10,204 (0.21%) Czech FBC patients compared to 1/3250 (0.03%) controls (p = 0.04) and in 4/3639 (0.11%) FBC patients from an independent German dataset. In addition, we found this variant in 5/2966 (0.17%) Czech (but none of the 443 German) ovarian cancer patients, three of whom developed early-onset tumors.

Based on these observations, we classified this variant as likely pathogenic.

## Highlights

• Deep intronic *CHEK2* variant c.1009-118_1009-87delinsC causes splice acceptor shift.
• The aberrant transcript p.Tyr337PhefsTer37 is subjected to nonsense-mediated decay.
• The variant was significantly enriched in breast cancer patients compared to controls.
• Carriers developed early-onset ER + breast cancer, a signature of *CHEK2* mutations.
• Our study supports that the c.1009-118_1009-87delinsC variant is likely pathogenic.

## 1 Introduction

The *CHEK2* (checkpoint kinase 2) gene encodes a nuclear serine/threonine-protein kinase CHK2, which is one of the key mediators of cellular response to various stress stimuli [1]. Its heterozygous germline pathogenic variants (GPV) confer a moderate breast cancer (BC) risk with OR = 2.47 and 2.54, respectively [2,3]. At the same time, *CHEK2* GPV are also associated with increased risk of multiple other cancer types including colorectal, thyroid, pancreatic, kidney or hematological malignancies [[4], [5], [6], [7], [8], [9]], reviewed in Ref. [10]. Compared to heterozygotes, carriers of homozygous or compound heterozygous *CHEK2* GPV have substantially higher cancer risk, develop tumors at younger age, but otherwise do not develop other clinical symptoms [11,12].

Importantly, *CHEK2* is the second most frequently altered BC predisposition gene in female BC patients of European ancestry, surpassed by *BRCA2* and followed by *BRCA1* [2,3]. Frequency of *CHEK2* GPV (truncations, splicing alterations, and large copy number variations) is approximately 1.1–1.3% in unselected female BC patients from Europe and the USA [2,3]. Additionally, 0.5% of BC patients may be carriers of rare missense *CHEK2* GPV [13].

In this study, we have identified previously unreported frequent deep intronic *CHEK2* GPV, characterized its effect at the RNA level, and provided evidence for its contribution to increased BC risk.

## 2 Patients and methods

**Identification of c.1009**–**118_1009**–**87delinsC *CHEK2* variant.** We have re-analyzed next generation sequencing (NGS)-based anonymized data from 10,204 female BC and 2966 ovarian cancer (OC) Czech patients clinically tested using the CZECANCA panel (including *CHEK2*) within the Czech consortium of diagnostic laboratories (www.czecanca.cz) [[14], [15], [16], [17]]. We have specifically searched for deep intronic germline *CHEK2* (NM_007194.4) variants localized outside the canonical intronic splice sites. Impact of identified *CHEK2* variant on pre-mRNA processing was analyzed by CZECANCA panel-based total RNA sequencing as described previously [18,19]. RNA was extracted from peripheral blood leukocytes with/without nonsense-mediated decay (NMD) inhibition with cycloheximide (final concentration 200 μg/mL) for 4 h. For variant burden analysis (two-sided Fisher's exact test), we used data from 3250 unselected Czech female population-matched controls (PMC) analyzed by the same NGS approach.

Variant frequency was independently assessed by analysis of data from 3639 and 443 German BC and OC patients, respectively (described previously [20]) using GATK HaplotypeCaller variant calling. As the variant's localization was outside (but close to) the corresponding sequencing target region of the TruRisk® panel applied (Human hg19 chr22:29092810–29093050), sufficient read depth (≥30) at chr22:29093063 was ensured using the samtools mpileup utility prior to variant calling.

All participants gave informed consent to germline genetic testing approved by ethics committees.

## 3 Results

**Fig. 1:**
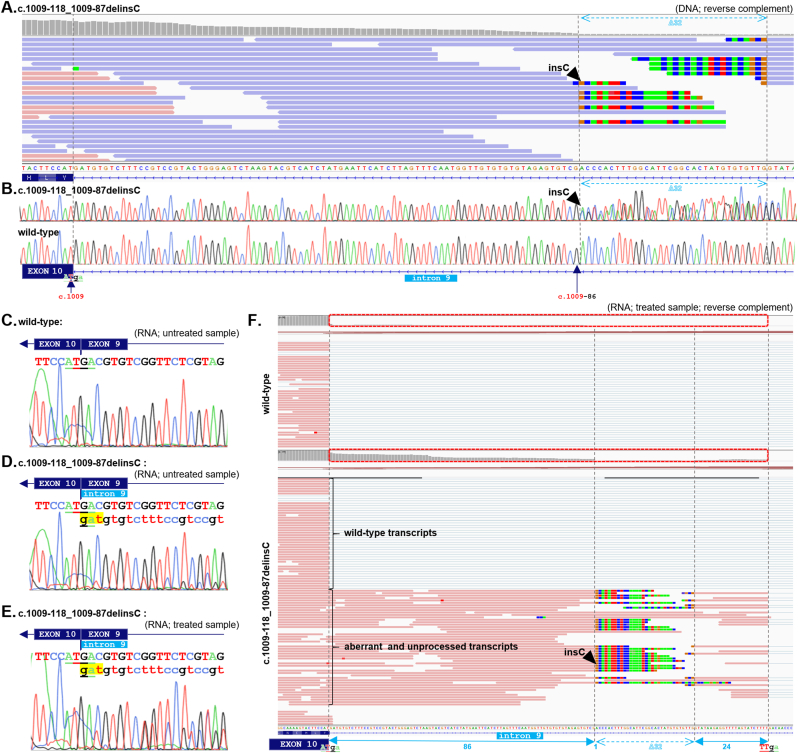
**Characterization of the c.1009**–**118_1009**–**87delinsC *CHEK2* variant.** (**A.**) NGS-based DNA sequencing visualized in the Integrative Genomics Viewer (IGV). The dashed gray lines indicate the deletion borders, and the dashed blue arrow denotes the deletion of 32bp following the nucleotide c.1009-86 at the 3′-end of intron 9 with the insertion of cytosine. (**B.**) DNA Sanger sequencing of the variant and wild-type samples. (**C-E.**) RNA (cDNA) Sanger sequencing of wild-type sample (C.), variant sample without (D.) and with (E.) NMD inhibition (cycloheximide), showing the increase of aberrant splicing variant signal peaks after NMD inhibition. (**F.**) RNA panel NGS from a wild-type control (top) and from a carrier of the c.1009-118_1009-87delinsC variant after NMD inhibition (bottom). Note the difference between wild-type and variant RNA in number (coverage) of intronic retentions (red dashed-line boxes) resulting from the aberrant pre-mRNA splicing. Solid blue arrows indicate sequencing context of aberrant reads showing 86b retained from intron 9, interrupted by 32b deletion replaced by single cytosine insertion, followed by 24b from intron 9: r.1008_1009ins1009-142_1009-1del1009-118_1009-87insC. We hypothesize that the reassembled primary transcript enhances the pre-existing alternative acceptor splice site TT|ga in intron 9, which precedes the canonical acceptor splice site upstream of exon 10. **Note:** The IGV visualizes the *CHEK2* sequence in a reverse complement according to the *CHEK2* gene reverse orientation on the chromosome 22. (For interpretation of the references to color in this figure legend, the reader is referred to the Web version of this article.)

By bioinformatic re-analysis of panel NGS data from Czech BC/OC patients, we identified previously unreported deep intronic *CHEK2* variant localized 87bp apart from the 3′-end of intron 9. It consisted of a 32bp deletion replaced by a single cytosine: c.1009-118_1009-87delinsC (Fig. 1A), which we confirmed by Sanger sequencing (Fig. 1B). Sanger sequencing and NGS-based total RNA analysis from available variant carriers' samples showed its clear effect on CHEK2 pre-mRNA splicing. Compared to the wild-type mRNA sequence (Fig. 1C), the variant allele leads to the use of upstream alternative splice acceptor at position c.1009-142 with consequent retention of terminal part of intron 9 at the beginning of exon 10 (r.1008_1009ins1009-142_1009-1del1009-118_1009-87insC). At the protein level, this aberrant CHEK2 transcript results in premature termination of translation (p.Tyr337PhefsTer37). Importantly, proportion of this aberrant CHEK2 transcript varied between 0.07 and 0.26 in RNA samples (Fig. 1D), which suggests either its NMD-mediated degradation or only partial effect of investigated variant on pre-mRNA splicing. NMD inhibition in samples from variant carriers resulted in significant increase of aberrantly spliced mRNA to the proportion >0.46 (Fig. 1E and F), confirming its partial degradation via NMD and indicating that majority (>90%) of variant allele generates aberrant transcripts.

The c.1009-118_1009-87delinsC variant was identified in 21/10,204 (0.21%) female BC cases (Table 1) and only in 1/3250 (0.03%) PMC (OR = 6.7; 95%CI 1.08–276.88, p = 0.04). Biallelic *CHEK2* inactivation was found in two BC patients (one of whom developed bilateral BC at the age of 33 years). Only one of all variant carriers had a co-occurring GPV in another BC predisposition gene (Table 1). All BC c.1009-118_1009-87delinsC variant carriers with known histology (18/21) developed ER-positive primary tumors, six developed double-primary cancers, and only two with known family cancer history (18/21) had negative or *CHEK2*-irrelevant family cancer history (Table 1). Additionally, we have identified higher frequency of c.1009-118_1009-87delinsC variant carriers also among OC cases (5/2966; 0.17%, Table 1) in comparison to controls, however, the difference was not statistically significant (OR = 5.49; 95%CI 0.62–259.25; p = 0.11).

**Table 1:** **Clinical and histopathological characteristics of breast cancer** (top and middle) **and ovarian cancer** (bottom) **patients carrying germline c.1009**–**118_1009**–**87delinsC *CHEK2* variant.**

Patient number	Age at diagnosis (years)	ER	PR	HER2	Histology	Double primary tumor (age; years)	Family cancer history
***CARRIERS WITH BREAST CANCER – Czech cohort (10,204 patients)***							
02_655a22	29	+	–	–	DCIS		CRC
02_748a22	30	+	+	+	L		CRC, LC
01_B2542^#1^	33	+/+	+/+	+/+	D/L	BC (33)	BC
04_469818	34	n.a.	n.a.	n.a.	n.a.		n.a.
07_B7594^#2^	39	+	+	+	D		CRC, PrC
04_1550a16	41	+	+	–	D		BC, GC
02_1101a20	44	+	–	+	D		LC
11_200451	44	+	+	+	D		PaC, PrC, RCC
07_B5877	45	+	+	+	D		neg.
04_1728421^#3^	45	n.a.	n.a.	n.a.	n.a.		BC
02_1339a20	46	+	+	–	DCIS	GC	BC
01_PKM488	47	+	+	+	D		CRC, PrC
02_150a22	48	+/−	+/−	−/−	L/D	BC (58)	BC
04_1230219	49	+	+	–	D		OC, CRC, GC
01_PKM165	53	+	–	n.a.	DCIS	Hem (51)	CRC, GC
02_1080a19	58	n.a.	n.a.	n.a.	DCIS	Hem (40)	BC
04_1658119	58	+	+	+	D		BC, PrC, LC
04_972619	60	+	+	–	D	CRC (71); Hem (60)	BC, OC
04_484419	62	+	+	–	D		BC
01_PKM4433	65	+	+	+	D		n.a.
01_PKM3591	71	+	–	–	D		n.a.
***CARRIERS WITH BREAST CANCER – German cohort (3639 patients)***							
58–10	45	+/+	+/−	+/−	D/L	BC	OC
13–24	47	n.a.	n.a.	n.a.	n.a.		BC
76–32	48	+	–	–	n.a.		BC
15–6	56	n.a.	n.a.	n.a.	n.a.		BC
***CARRIERS WITH OVARIAN CANCER – Czech cohort (2966 patients)***							
04_39420	24	×	×	×	DYS		UBC
01_B1231	32	×	×	×	HGS		BC
08_BS670a19	34	×	×	×	n.a.	RCC (24)	OC, CRC, GC, PrC, RCC, BT, OS, Hem
02_1167a20	51	×	×	×	HGS		GC
04_355619	65	×	×	×	PAP		neg.

**Legend:** Individuals with additional pathogenic germline variant in ^#1^*CHEK2*: c.277del (p.Trp93GlyfsTer17); ^#2^*BRCA2*: c.673_676del (p.Thr225LeufsTer4); ^#3^*CHEK2*: c.1009-118_1009-87delinsC homozygote.

**BC** – breast cancer, **BT** – brain tumor, **CRC** – colorectal cancer, **D** – ductal BC, **DCIS** – ductal BC (in situ), **DYS** – dysgerminoma, **ER** – estrogen receptor, **GC** – gastric cancer, **Hem** – hematological malignancies, **HER2** – HER2 receptor, **HGS** – high-grade serous OC, **L** – lobular BC, **LC** – lung cancer, **n.a.** – not available, **neg** – negative, **OC** – ovarian cancer, **OS** – osteosarcoma, **PaC** – pancreatic cancer, **PAP** – papillary adenocarcinoma, **PR** – progesterone receptor, **PrC** – prostate cancer, **RCC** – renal cell carcinoma, **UBC** – urinary bladder cancer, + – positive, - – negative, × – not applicable.

Analysis of independent German dataset revealed four carriers of the c.1009-118_1009-87delinsC variant among 3639 BC patients (0.11%) and in 0/443 German OC patients. The frequency in German BC patients was significantly higher than in European (non-Finnish) from gnomAD_v3 controls (as 22-28697075-GGGTGAAACCGTAAGCCGTGATACACACAAC-G; 2/34,018; 0.006%; p = 0.001).

## 4 Discussion and conclusions

We have characterized a recurrent germline *CHEK2* deep intronic variant c.1009-118_1009-87delinsC and showed that it leads to the formation of aberrantly spliced CHEK2 mRNA partially subjected to NMD, and encodes a functionally impaired prematurely terminated protein. Furthermore, we showed that this variant was overrepresented in female BC patients with frequency similar to other two Slavic founder splicing *CHEK2* GPV c.444+1G > C and c.846 + 4_846+7del described in Czech population previously [21]. Importantly, germline c.1009-118_1009-87delinsC positive BC patients had characteristics typical for *CHEK2* GPV carriers (ER-positive tumors, cancer multiplicity, and positive family history of cancer) [22]. Moreover, identified *CHEK2* variant was associated with increased risk of female BC development (OR = 6.7). On the other hand, we believe that the risk level was overestimated due to lower number of PMC and may be similar as for *CHEK2* pathogenic truncations (OR = 2.47–2.54) or missense variants (OR = 2.83) [2,3,13]. There is generally conflicting evidence for OC predisposition and *CHEK2* GPV [13,21]. Nevertheless, we recurrently observed c.1009-118_1009-87delinsC in OC patients, including early-onset patients who may otherwise represent a specific OC subgroup with an unusually low proportion of GPV in established cancer predisposition gene [23].

In conclusion, the *CHEK2* variant c.1009-118_1009-87delinsC results in an aberrant mRNA transcript containing premature termination codon (p.Tyr337PhefsTer37), producing a functionally impaired CHK2 kinase isoform (ACMG code PS3 - moderate) [13]. The mRNA transcript is partially subjected to NMD [24,25]. The variant was significantly enriched in BC patients (ACMG code PS4 - strong) with a phenotype typical for known *CHEK2* GPV. This led us to classify the c.1009-118_1009-87delinsC variant as likely pathogenic. Further studies are necessary to confirm its clinical implications and to establish its prevalence in other populations. Our study highlights the critical importance to focus on intronic regions beyond the canonical ±1/2 splice sites within the search for RNA splicing affecting GPV.

## Funding

This work has been supported by the grant projects of the 10.13039/501100003243Ministry of Health of the Czech Republic [NU23-03-00150, NU20-03-00283, NU20-03-00016, NU20-03-00285, DRO-VFN-64165]; 10.13039/100007397Charles University projects [COOPERATIO, SVV260631, UNCE/24/MED/022]; and the Ministry of Education Youth and Sports of the Czech Republic grant [Programme EXCELES, ID Project No. LX22NPO5102 - Funded by the European Union – Next Generation EU, and The National Center for Medical Genomics LM2023067]; 10.13039/501100005972German Cancer Aid [70114178].

## Declaration of competing interest

All authors have no conflicts of interest to declare.

## Ethics approval

All individuals provided written informed consent with genetic testing approved by the Ethics Committees of participating institutions and the study was performed in accordance with the Declaration of Helsinki.

## CRediT authorship contribution statement

**Petra Zemankova:** Writing – review & editing, Writing – original draft, Visualization, Validation, Formal analysis, Conceptualization. **Marta Cerna:** Writing – review & editing, Writing – original draft, Visualization, Investigation. **Klara Horackova:** Investigation, Validation, Writing – review & editing. **Corinna Ernst:** Formal analysis, Writing – review & editing. **Jana Soukupova:** Data curation, Investigation, Writing – review & editing. **Marianna Borecka:** Investigation, Resources, Writing – review & editing. **Britta Blümcke:** Writing – review & editing, Data curation, Resources. **Leona Cerna:** Writing – review & editing, Data curation. **Monika Cerna:** Writing – review & editing, Data curation. **Vaclava Curtisova:** Writing – review & editing, Resources. **Tatana Dolezalova:** Writing – review & editing, Resources. **Petra Duskova:** Writing – review & editing, Data curation. **Lenka Dvorakova:** Writing – review & editing, Resources. **Lenka Foretova:** Writing – review & editing, Resources. **Ondrej Havranek:** Writing – review & editing, Resources. **Jan Hauke:** Writing – review & editing, Funding acquisition, Data curation. **Eric Hahnen:** Writing – review & editing, Resources. **Miloslava Hodulova:** Writing – review & editing, Resources, Investigation. **Milena Hovhannisyan:** Investigation, Resources, Writing – review & editing. **Lucie Hruskova:** Data curation, Writing – review & editing. **Marketa Janatova:** Investigation, Resources, Writing – review & editing. **Maria Janikova:** Data curation, Writing – review & editing. **Sandra Jelinkova:** Writing – review & editing, Resources, Investigation. **Pavel Just:** Writing – review & editing, Resources, Investigation. **Marcela Kosarova:** Writing – review & editing, Resources. **Monika Koudova:** Data curation, Writing – review & editing. **Vera Krutilkova:** Data curation, Writing – review & editing. **Eva Machackova:** Data curation, Investigation, Writing – review & editing. **Katerina Matejkova:** Writing – review & editing, Resources. **Renata Michalovska:** Writing – review & editing, Resources. **Adela Misove:** Writing – review & editing, Resources, Investigation. **Petr Nehasil:** Formal analysis, Resources, Writing – review & editing. **Barbora Nemcova:** Resources, Writing – review & editing. **Jan Novotny:** Resources, Writing – review & editing. **Ales Panczak:** Resources, Writing – review & editing. **Pavel Pesek:** Investigation, Resources, Writing – review & editing. **Ondrej Scheinost:** Resources, Writing – review & editing. **Drahomira Springer:** Resources, Writing – review & editing. **Barbora Stastna:** Resources, Writing – review & editing. **Viktor Stranecky:** Data curation, Writing – review & editing. **Ivan Subrt:** Resources, Writing – review & editing. **Spiros Tavandzis:** Data curation, Writing – review & editing. **Eva Tureckova:** Investigation, Resources, Writing – review & editing. **Kamila Vesela:** Writing – review & editing, Resources. **Zdenka Vlckova:** Resources, Writing – review & editing. **Michal Vocka:** Writing – review & editing, Resources, Data curation. **Barbara Wappenschmidt:** Writing – review & editing, Resources. **Tomas Zima:** Writing – review & editing, Resources. **Zdenek Kleibl:** Writing – review & editing, Writing – original draft, Visualization, Supervision, Funding acquisition, Conceptualization. **Petra Kleiblova:** Writing – review & editing, Writing – original draft, Supervision, Funding acquisition, Conceptualization.
